# Potential of the SPY intraoperative perfusion assessment system to reduce ischemic complications in immediate postmastectomy breast reconstruction

**DOI:** 10.1186/1750-1164-7-9

**Published:** 2013-07-23

**Authors:** Mohit Sood, Paul Glat

**Affiliations:** 1 Philadelphia College of Osteopathic Medicine, Cherry Hill, New Jersey; 2 Eastern Regional Medical Center, Inc, Philadelphia, Pennsylvania

## Abstract

**Background:**

The quality and viability of mastectomy flaps remain a central challenge in reconstructive surgery, particularly for immediate breast reconstruction. Insufficient perfusion in tissue flaps is a leading cause of early complications following reconstructive procedures, and clinical judgment alone is not completely reliable for the assessment of flap viability. Accurate and reliable intraoperative methods for assessment of tissue perfusion are needed to help surgeons identify tissue at risk for ischemia and necrosis, thereby allowing for maneuvers to improve tissue flap viability.

**Methods:**

This study evaluates the use of intraoperative laser angiography using the SPY System (LifeCell Corp., Branchburg, NJ) for the assessment of perfusion in mastectomy flaps for immediate breast reconstruction. The SPY System uses the contrast agent indocyanine green, which has an excellent safety profile and pharmacokinetics that allow for repeat evaluations during the same surgical procedure. In recent work, the SPY System has demonstrated high sensitivity and specificity for detection of tissues at risk for ischemia and necrosis during reconstructive surgery. Using a retrospective, chart-review design, the authors compared consecutive cases of immediate breast reconstruction using a prosthesis, before and after implementation of the SPY System.

**Results:**

Ninety-one subjects were included in the analysis: 52 prior to SPY (Pre-SPY) and 39 after implementation of SPY (Post-SPY). Baseline characteristics were similar between the groups. Both groups had high rates of comorbidities, chemotherapy, and radiation therapy. The rate of postoperative complications was two-fold higher in the Pre-SPY group compared to the Post-SPY group (36.5% vs. 17.9%); this difference was of borderline significance (P = 0.0631). However, mean number of repeat visits to the OR per patient was significantly higher in the Pre-SPY group (1.21 ± 1.47 vs. 0.41 ± 0.71; P = 0.0023). Of the seven patients with complications in the Post-SPY group, five were identified by SPY as having poor flap perfusion; none were identified by clinical judgment alone.

**Conclusions:**

This study suggests that the SPY System can contribute to reduced ischemia-related complications in a population of women undergoing immediate breast reconstruction following mastectomy for breast cancer.

## Background

Approximately 40% of women who undergo mastectomy for breast cancer will elect to have breast reconstruction [1]. The optimal approach to postmastectomy breast reconstruction continues to evolve. Patient-, surgeon-, and disease-related factors often influence the timing and type of reconstruction selected. For appropriate patients, U.S. practice has evolved to include immediate reconstruction with one-stage, direct-to-implant procedures or two-stage procedures with insertion of tissue expanders (TE) followed by replacement with permanent implant.

Immediate reconstructive procedures have been made more feasible through surgical approaches that preserve the native skin envelope, including skin-sparing and nipple-sparing mastectomy. Key issues in immediate reconstructive procedures include coverage of the implant, the use of appropriate TE, and the quality and viability of tissue flaps.

In direct-to-implant procedures, local tissues and/or reinforcing prostheses must be employed to provide adequate coverage of the implant [2]. Recently, the use of human acellular dermal matrix (HADM; AlloDerm [LifeCell Corp., Branchburg, NJ]) has gained favor for use in both direct-to-implant and TE breast reconstructions [3-7]. Characteristics of HADM that support its use in this setting include pliability, strength, and the promotion of cellular repopulation and revascularization, which may increase its ability to resist or overcome infection [8-10].

The quality and viability of mastectomy flaps is a central challenge in reconstructive surgery. For immediate reconstructive procedures, the flaps must be of sufficient size to cover the TE or implant and must maintain adequate blood flow to prevent necrosis or other complications. Indeed, a leading cause of early complications following reconstructive procedures is insufficient perfusion in tissue flaps [11-14]. Furthermore, in the postmastectomy setting, chemotherapy, radiation therapy, and other variables are known to increase the risk for impaired wound healing and other complications [15,16]. The management of complications, particularly those requiring return to the OR, is a major cost driver for breast reconstructive procedures [17,18], for both the patient and the hospital.

For these reasons, surgeons have evaluated and employed a number of methods to assess tissue perfusion in the intraoperative setting. An ideal technique would allow for accurate identification of perforating vessels and their perfusion zones, assessment of tissue perfusion, and identification of tissue at risk for necrosis. Clinical judgment is the most widely used method for evaluating blood supply and tissue viability [19]. However, evidence suggests that clinical assessment alone is not completely reliable for assessment of tissue perfusion [20,21].

Technologies evaluated for assessment of tissue perfusion in the intraoperative setting include hand-held Doppler, laser Doppler flowmetry, and fluorescein angiography [22-26]. Each of these methods suffers from significant limitations. Evidence suggests that Doppler ultrasound is operator-dependent and provides limited accuracy for flap design [22-27]. Laser Doppler is cumbersome, may underestimate flap survival, and may have poor ability to detect perforators [23,24]. Fluorescein is limited by the long half life of the contrast medium, which prevents re-evaluation during the intraoperative period, and the potential for false-positive findings [25,28,29].

In recent years, some surgeons have adopted indocyanine green (ICG) angiography, using the SPY intraoperative perfusion assessment system (LifeCell Corp., Branchburg, NJ), for assessment of tissue perfusion in mastectomy flaps [13,30-32]. The fluorescence agent ICG has a short half life, binds strongly to plasma proteins, and has an excellent safety profile, allowing for rapid clearance from tissues and the performance of repeat evaluations during the same surgical procedure [33]. Recent work suggest that the SPY system also provides greater accuracy compared to fluorescein or clinical judgment for prediction of mastectomy flap necrosis [34].

The current retrospective, chart-review study was undertaken to evaluate the impact of the adoption of the SPY system on complications following immediate TE- or implant-based breast reconstruction with HADM following mastectomy.

## Methods

The study was performed at a single institution in a retrospective, chart-review design. All surgeries were performed by the authors. The study compared consecutive cases of immediate breast reconstruction using prosthesis before (January 1, 2009 – April 1, 2011) and after (April 1, 2011 – April 30, 2012) implementation of the SPY intraoperative perfusion assessment system for reconstructive procedures. This study was granted exemption from IRB review based on its retrospective, chart-review design.

Subjects were identified through analysis of hospital records. Consecutive patients undergoing immediate breast reconstruction with a prosthesis following mastectomy for treatment of breast cancer were included. Subjects treated with TE and/or implants were included. Subjects treated with delayed procedures or flap-based reconstructions were excluded. All breast reconstructions in this study were performed using HADM. Data were collected for subject age, tumor stage, surgery, tobacco use, comorbidities, implant size, number of repeat trips to OR, and complications.

All implant reconstructions were performed using a standard sub-pectoral implant position. The pectoralis major was separated from its inferomedial costal attachments. Once completed, the implant was soaked in antibiotic solution and placed into the pocket. For inferior coverage, HADM was secured to the edge of the cut pectoralis and the inframammary shelf. The wounds were thoroughly irrigated and drains were placed into the axilla and inferior aspect of the wound. Mastectomy flaps were routinely closed in a layered fashion with interrupted 3.0 polydioxanone deep dermal suture and 4.0 monocryl intradermal closure.

Once SPY became available at the authors’ institution, the device was used in addition to clinical judgment to assess perfusion in mastectomy flaps after the general surgeon completed the mastectomy and prior to any reconstructive procedures. Briefly, 3 cc ICG were administered by IV push, followed by 10 cc normal saline flush. Fluorescence images were captured using the digital camera on the SPY System and archived for reference. Areas of low fluorescence, indicating limited perfusion in the flap, were noted, and maneuvers were performed to improve perfusion according to surgeon judgment and preference. These maneuvers included resection of skin in potentially ischemic areas and/or reduction of implant volume.

Analysis of the data was performed using chi square and Fisher’s exact test for categorical variables and unpaired two-tailed t-test for continuous variables.

## Results

A total of 95 consecutive subjects met the inclusion criteria for this study, 54 prior to (Pre-SPY) and 41 after implementation of SPY (Post-SPY). Two subjects in the Pre-SPY group were excluded based on flap reconstructions; two subjects in the Post-SPY group were excluded, one for flap reconstruction and one for delayed reconstruction. Therefore, the final sample included 52 subjects in the Pre-SPY group and 39 in the Post-SPY group. There were 24 unilateral reconstructions and 28 bilateral reconstructions, totaling 80 operated breasts. Demographic and baseline characteristics of the subjects are listed in Table 1. There were no significant differences between groups in terms of age, presence of comorbidities, history of smoking, tumor status, or cancer therapy (chemotherapy, hormonal therapy, radiation therapy).

**Table 1:** Baseline characteristics of subjects

	**Pre-SPY**	**Post-SPY**	P-value*
N	52	39	
Age, mean (SD)	52 (7.9)	51 (8.6)	0.5666
Any smoking, N (%)	13 (25.5)**	14 (35.9)	0.3550
Comorbidities present, total, N (%)	40 (78.4)**	24 (61.5)	0.1020
Diabetes, N (%)		3 (7.7%)	1.000
Obesity, N (%)	5 (9.6%)	2 (5.1%)	1.000
	2 (3.8%)		
Tumor status, N (%)			
T1	21 (40.4)	10 (25.6)	NS^†^
≥T2	26 (50.0)	24 (46.2)	
N0	27 (51.9)	19 (48.7)	
≥N1	21 (40.4)	15 (28.8)	
M0	42 (80.8)	32 (82.1)	
M1	5 (9.6)	2 (5.1)	
DCIS	5 (9.6)	2 (5.1)	
Other	1 (1.9)	3 (7.7)	
Therapy			
Any chemotherapy, N (%)	40 (76.9)	25 (64)	NS^‡^
Any hormonal therapy, N (%)	29 (55.8)	18 (46.1)	
Any radiation, N (%)	27 (51.9)	17 (43.6)	
No therapy, N (%)	4 (7.7)	5 (12.8)	

* Unpaired two-tailed t-test.

** Data missing for one subject.

^†^ No significant interaction by group and tumor status: T1 vs. >T1, N0 vs. >N0, or M0 vs. M1, Fisher’s exact test.

^‡^ No significant interaction by group and each therapy vs. absence of each therapy, Fisher’s exact test.

DCIS: ductal carcinoma in situ.

NS: Not significant at P < 0.05.

Note: identified comorbidities included hypertension, diabetes, obesity, asthma, hyperlipidemia, thyroid disease, gastroesophageal reflux disease, osteoporosis, irritable bowel syndrome, thyroid disease, arthritis, endometriosis, chronic bronchitis, sinusitis, hepatitis B, deep vein thrombosis, rheumatic fever, lupus, obstructive sleep apnea, sickle cell disease, anxiety, and depression.

There were no significant differences between groups with regard to reconstructive modality (Table 2). Similar proportions of each group underwent bilateral (Pre-SPY, 53.8%; Post-SPY, 59%) and unilateral (Pre-SPY, 46.2%; Post-SPY, 41.0%) breast reconstruction. There were no significant differences between groups in the use of TE or implants. Mean implant size was greater in the Post-SPY group (mean 575.9 vs. 519.8 cc), but this difference did not reach statistical significance (P = 0.074).

**Table 2:** Surgical treatments and outcomes, by group

	**Pre-SPY**	**Post-SPY**	**P-value**
Bilateral, N (%)	28 (53.8)	23 (59.0)	0.674^†^
Unilateral, N (%)	24 (46.2)	16 (41.0)	
Implant, N (%)	49 (94.2)*	22 (56.4)	0.215^†^
Tissue expander, N (%)	22 (42.3)*	17 (43.6)	
Implant size, mean cc (SD)	519.8 (164.7)	575.9 (140.5)	0.074**
Total complications, N (%)	19 (36.5)	7 (17.9)	0.0631^†^
Flap necrosis	9 (17.3)	4 (10.3)	
Capsular contracture	5 (9.6)	0 (0.0)	
Cellulitis	2 (3.8)	1 (2.6)	
Hematoma	0 (0.0)	1 (2.6)	
Extrusion of TE	1 (1.9)	0 (0.0)	
Displacement of implant	1 (1.9)	0 (0.0)	
Deflation of TE	1 (1.9)	0 (0.0)	
Other	0 (0.0)	1 (2.6)^‡^	
No. repeat OR visits, mean (SD)	1.21 (1.47)	0.41 (0.71)	0.0023**

*Includes multiple repeat procedures for individual patients.

**Unpaired t-test.

^†^Fisher’s exact test.

^‡^One incidental suture spitting noted during port placement.

The rate of complications was more than twice as high in the Pre-SPY group compared to the Post-SPY group (36.5% vs. 17.9%; Table 2); this finding approached but did not reach statistical significance (P = 0.0631). The rate of flap necrosis was 17% in the Pre-SPY group compared to 10% in the Post-SPY group (P = 0.383). Nineteeen (36.5%) patients in the Pre-SPY group developed complications. These complications included flap necrosis (9 patients), capsular contraction (5 patients), cellulitis (2 patients), extrusion of the TE (1 patient), displacement of the implants (1 patient), and deflation of the TE (1 patient). Seven (17.9%) patients in the Post-SPY group developed complications. Five of these patients were identified by SPY as having poor flap perfusion (see Table 3); none were identified by clinical assessment alone. For the five patients in this group identified by SPY as having poor perfusion, surgical maneuvers were performed to prevent necrosis, including debrediment of non-perfused tissue, deflation of the tissue expander to improve blood flow, and the use of transdermal nitroglycerin ointment to treat vasospasm. Complications for these seven patients in the Post-SPY group included necrosis of skin flaps and/or nipple (4 patients), hematoma (1 patient), cellulitis and seroma (1 patient), and incidental suture spitting noted during port placement (1 patient). Five of these patients’ complications (necrosis [n = 4] and cellulitis [n = 1]) could relate to poor perfusion. The others (hematoma and suture spitting) are unlikely to be related to perfusion.

**Table 3:** Number of patients with complications, by ischemia assessment method

**Assessment method**	**Patients with complications**
SPY	
Ischemia present, N (%)	5 (71.4)*
No ischemia detected, N (%)	2 (28.6)**
Clinical assessment	
Ischemia present, N (%)	0 (0.0)
No ischemia detected, N (%)	7 (100)
Total patients with complications, N (%)	7 (100)

Note: percent of patients with complications by assessment method and presence of ischemia/total number of patients with complications (N = 7).

* Complications in these patients included flap necrosis (n = 4) and suture failure (n = 1).

** Complications in these patients included hematoma (n = 1) and cellulitis and seroma (n = 1).

Two additional analyses were performed based on the potential relationship between complications and ischemia. The first compared the combined incidence of flap necrosis and cellulitis between groups (Pre-SPY n = 11, Post-SPY n = 5); this comparison was not statistically significant (P = 0.407). Based on evidence that ischemia could contribute to capsular contracture, the second analysis compared the combined incidence of flap necrosis, cellulitis, and capsular contracture between groups (Pre-SPY n = 16; Post-SPY n = 5); this difference was statistically significant (P = 0.0492).

In the Pre-SPY group, 32 patients (61.5%) required return visits to the OR (total of 63 visits); in the post-SPY group, 11 patients (28.2%) required return visits to the OR (total of 14 visits). Mean number of repeat visits to the OR per patient was significantly higher in the Pre-SPY group: 1.21 ± 1.47 vs. 0.36 ± 0.66 (95% confidence interval 0.3477 – 1.3523; P = 0.0011). The most common procedure performed on return to the OR in the Pre-SPY group was revision to treat dehiscence or necrosis (10 patients), followed by implant exchange (8 patients), capsular contracture (5 patients), minor revisions (4 patients), and other procedures (1 patient). The most common procedures performed on return to the OR in the Post-SPY group were implant exchange (4 patients) and treatment of necrosis (4 patients); one patient each was treated for cellulitis, hematoma, and suture spitting.

**Figure 1:**
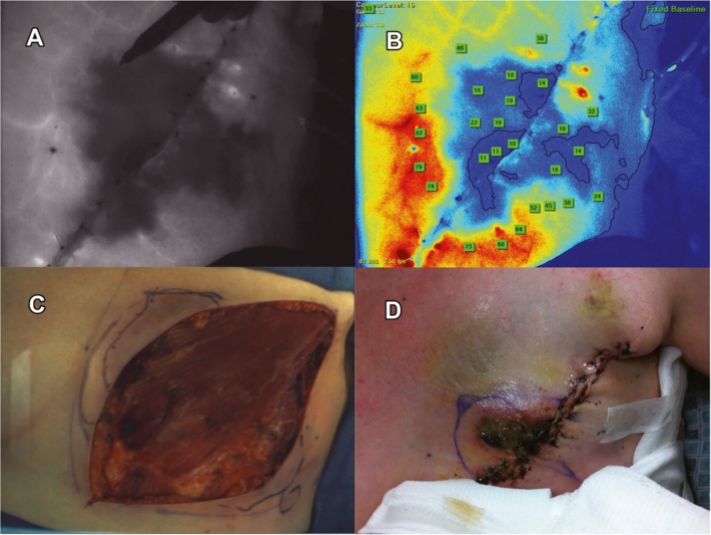
**Illustration of the use of the SPY System for evaluation of tissue perfusion in a mastectomy flap.** The black-and-white fluorescence image **(Panel A)** shows large dark area of minimal fluorescence, reflecting poor perfusion, surrounding the incision; the surgeon’s pen can be seen tracing the outline of this region. **Panel B** is a colorized version of the same image, showing quantification of absolute fluorescence (numbers in boxes); darker colors represent areas of lower fluorescence signal. The incision prior to reconstruction is shown in **Panel C**; areas of poor perfusion identified by SPY are noted in blue-pen outlines superior and inferior to the incision. In this case, clinical judgment of tissue viability (including appearance of skin and presence of bleeding at tissue edge) was favored over SPY findings, and the regions of poor perfusion noted on SPY were left intact. The post-operative result **(Panel D)** shows necrosis superior to the incision, corresponding to the region of poor perfusion identified by SPY.

**Figure 2:**
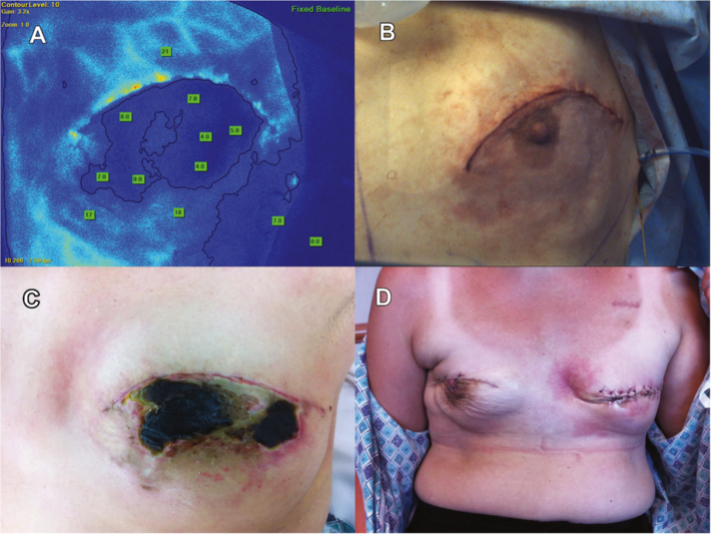
**Use of SPY to identify areas of poor perfusion prior to reconstruction.** The colorized SPY image of the left breast **(Panel A)** shows a region of low fluorescence (dark blue), corresponding to the nipple-areola complex (NAC). Numbers reflect quantification of absolute fluorescence values. Based on clinical judgment of tissue viability (including appearance of skin and presence of bleeding at tissue edge), the area identified by SPY was not removed. Following reconstruction **(Panel B)**, the NAC appears dusky inferior to the incision. Ultimately, the NAC region identified by SPY as having poor perfusion developed necrosis **(Panel C)** and required return to the OR for debridement and removal of the NAC **(Panel D)**.

Following implementation of SPY, perfusion in the mastectomy flaps was assessed both by SPY imaging and clinical judgment (see Table 4). Rates of identification of ischemia by clinical assessment and SPY were highly significantly different (P < 0.0001). In the Post-SPY sample, only one patient (2.6%) was determined by clinical assessment to have poor perfusion in the mastectomy flaps. Conversely, SPY imaging identified poor flap perfusion in 20 patients (51.3%), including the one patient identified by clinical assessment. Most of these patients (N = 18, 90%) were managed by resection of skin in the affected area; implant volume was decreased in nine (45%) patients. Selected cases in which clinical judgment of tissue viability was favored over SPY findings of poor perfusion are illustrated in Figures 1 and 2.

**Table 4:** Presence of ischemia in Post-SPY sample, by method of assessment

	**Ischemia present**		Management*		P-value**
	**Yes**	**No**	**Skin resection**	**Decrease implant volume**	
SPY, N (%)	20 (51.3)	19 (48.7)	18 (90)^†^	9 (45)^†^	<0.0001
Clinical judgment, N (%)	1 (2.6)	38 (97.4)	1 (100)^†^	1 (100)^†^	

* Management of ischemia in patients with “yes” answer, by method of assessment.

** Fisher’s exact test, presence of ischemia: SPY vs. clinical judgment.

^†^ Management approach by percent of patients with ischemia (N = 20 SPY, N = 1 clinical judgment).

## Discussion

Optimizing outcomes of postmastectomy breast reconstruction requires exploration of surgical techniques, materials, and technologies designed to improve cosmesis, limit costs, and minimize complications. In particular, surgeons and patients will benefit from the application of techniques that can identify ischemia in tissue flaps, allowing surgeons the opportunity to perform operative revisions and prevent ischemia-related complications. We believe this is one of the first studies to compare outcomes before and after implementation of SPY in postmastectomy patients treated with immediate breast reconstruction.

This retrospective study examined the use of the SPY intraoperative perfusion assessment system for the evaluation of tissue perfusion in mastectomy flaps in consecutive patients undergoing immediate breast reconstruction. The results demonstrate a two-fold reduction in overall complication rate following the introduction of SPY (36.5% to 17.3%). Although this difference did not achieve statistical significance (P = 0.0631), it strongly suggests that the use of intraoperative methods to accurately determine tissue perfusion as an augment to clinical judgment can profoundly reduce the incidence of ischemia, flap necrosis, and related complications. The impact of these complications may also be evident in reduced patient quality of life and additional financial costs to the individual hospital and the overall healthcare system. These potential impacts are bolstered by the significant difference between groups with regard to repeat OR visits (1.21 vs. 0.36; P = 0.0023). The lower rate of repeat OR visits in the Post-SPY group could relate to reduced need for operative treatment of complications in this group.

Previous studies of SPY in breast reconstruction have reported a strong correlation between fluorescence pattern on SPY and postoperative necrosis [11,30,31,35,36]. One recent, prospective study compared clinical judgment, fluorescein, and SPY for evaluation of tissue perfusion in 32 patients undergoing TE-implant reconstruction [34]. In this study, SPY demonstrated a sensitivity of 90% and specificity of 50% for prediction of necrosis. These and other investigators have also undertaken analysis of fluorescence values as determined by a SPY software package in an attempt to identify an objective threshold for ischemic versus non-ischemic tissue [34,37]. Phillips et al. identified a threshold of 3.7 absolute perfusion units, below which SPY had a sensitivity of 90% and specificity of 100% for prediction of necrosis [34]. Validation of such findings could provide surgeons with objective determinants of tissue perfusion and the accurate prediction of tissue necrosis using SPY.

Reported complication rates following immediate breast reconstruction range from ~4% to ~60% [2,16,38-41]. The overall complication rates in the current study fit within previously reported ranges. The relatively high rate of complications described in this and other studies of breast reconstruction may relate to several factors. Examination of breast tissue suggests that the breast harbors multiple endogenous bacteria that may contribute to postoperative infection, and it has been proposed that the breast should be considered a clean-contaminated surgical site [42]. Other factors prevalent in the current and previous studies include a high rate of comorbidities and the use of cancer therapies, such as chemotherapy and radiation therapy, each of which has been shown to increase the risk for postoperative complications following breast reconstruction [2,43-45]. Independent risk factors for surgical site infection (SSI) that have been identified following TE breast reconstruction include larger breast size, previous irradiation, repeated implants, and delayed reconstruction [2,46]. In one study of immediate breast reconstruction, irradiation increased risk for complications four fold [2]. Patient characteristics associated with increased risk for postoperative complications include smoking, obesity, and diabetes, among others [47-49].

In the current study, 78.4% and 61.5% of subjects in the Pre- and Post-SPY groups, respectively, had comorbidities at the time of surgery, including obesity, smoking, and diabetes. Rates of chemotherapy (76.9% Pre-SPY, 64% Post-SPY) and radiation therapy (51.9% Pre-SPY, 43.6% Post-SPY) were also high in both groups. Therefore, a high rate of complications could be expected based on these patient- and treatment-related factors. The expected high rate of complications highlights the potential benefits and economic value of augmenting clinical judgment with the use of SPY, which halved the complication rate in this challenging patient population.

The choice of immediate versus delayed reconstruction may also influence outcomes [16,50]. Immediate reconstruction has the potential to reduce number of operations, costs, and the period of disfigurement for appropriate patients [51,52]. However, some research suggests higher complication rates associated with immediate reconstruction. One comparison of immediate and delayed breast reconstructions found a significantly higher rate of complications following immediate placement of a TE compared to delayed reconstruction (P = 0.008). Interestingly, capsular contracture was significantly more common following immediate (40.4%) compared to delayed reconstruction (17%; P < 0.001) [50].

Several complications, in addition to flap necrosis, may be associated with poor perfusion. These potential complications of ischemia, including infection (eg, cellulitis), poor wound healing, anastomotic thrombosis, and fat necrosis, have been described in the surgical literature [53-57]. Some evidence suggests that seemingly unrelated complications, such as capsular contracture, may also be influenced by ischemia. In the current study, capsular contracture was identified as a complication in 9.6% of subjects in the Pre-SPY group and 0% of the Post-SPY group. Identified risk factors for capsular contracture include clinical or subclinical infection, and bacterial colonization of breast implants and the TE pocket has been reported [58-61]. Therefore, it is possible that the use of SPY could contribute to a reduction in capsular contracture rates by allowing the surgeon to optimize tissue perfusion and minimize ischemia and necrosis in the flap. It is also possible that capsular contracture presents over a longer time frame, and the shorter follow up in the Post-SPY group has yet to encompass these events. One interesting development is the emergence of data suggesting that HADM can mitigate capsular contracture [2,6]. It is possible that the use of HADM in the current study contributed to a relatively low incidence of capsular contracture.

### Technical considerations

It became apparent that there was a learning curve associated with the use of SPY. Initially, the authors were reluctant to rely on SPY findings when they conflicted with clinical assessment. After evaluation of initial results using SPY, treating surgeons gave greater importance to SPY findings. This learning period lasted approximately three months (~April 1 – July 1, 2011). A total of 7 patients (2 with complications) in the Post-SPY group underwent initial operation during this learning period. Removal of these cases from the Post-SPY group identified a total of 32 patients, 5 (15.6%) of whom had complications. Comparison of this post-learning period SPY group to the Pre-SPY group demonstrated a significant difference in complication rates (36.5% vs. 15.6%; P = 0.0484).

The impact of this learning period is exemplified by specific patients in the Post-SPY group (see Figures 1 and 2). For example, one case performed soon after the introduction of SPY (June 17, 2011) was determined by clinical assessment to have adequate perfusion in the mastectomy flaps. However, SPY identified regions of low fluorescence, indicating poor tissue perfusion. In this case, the authors relied on clinical assessment over the SPY findings, and no revisions were made. The patient subsequently developed skin flap necrosis and TE extrusion one month later. This type of post-operative complication also results in increased costs to the hospital and reduced quality of life for the patient.

### Cost considerations

Postoperative complications can have a profound impact on total costs of care. One analysis of 949 women undergoing breast surgery reported that patients with SSI had crude median costs of $16,882, compared to $6,123 for uninfected patients [57]. On multivariate analysis, the cost attributable to SSI for postmastectomy breast reconstruction procedures was $4,091. With an estimated $14 billion spent annually in the U.S. on care for patients with breast cancer, it is likely that even modest reductions in complication rates following reconstructive surgery could have substantial impact on the overall cost to individuals, payers, and society [62].

Several groups have suggested that immediate reconstruction may provide cost savings over delayed procedures [52,60]. A systematic review study analyzed cost data for the use of HADM in breast reconstruction and found that direct-to-implant reconstructions with HADM were associated with lower costs compared to two-stage, non-HADM procedures [63].

### Limitations

The main limitations of this study are its retrospective, non-randomized design and small sample size. Future studies would benefit from prospective data collection, randomization of subjects, and sample size powered to detect differences in rates of specific complications.

## Conclusions

This study provides initial evidence of the effectiveness of the SPY System in reducing ischemia-related complications in a population of women undergoing immediate breast reconstruction following mastectomy for breast cancer.

To our knowledge, this is one of the first reports to compare complication rates before and after implementation of SPY in this patient population. These initial findings must be replicated in larger, prospective studies to provide surgeons with valuable guidance regarding the potential of SPY to predict tissue necrosis in breast reconstruction procedures and illustrate the potential costs savings to hospitals and improved quality of life for breast cancer patients undergoing reconstruction. Future outcome analyses should also include financial analysis to determine if improved intraoperative perfusion assessment reduces total costs through the avoidance of complications requiring additional care.

## Consent

Written informed consent was obtained from patients for the publication of this report and accompanying images.

## Competing interests

Dr. Sood has nothing to disclose. Dr. Glat is on the Speakers’ Bureau for LifeCell Corp.

## Authors’ contributions

PG and MS carried out surgical studies and contributed to preparation of the manuscript. Both authors read and approved the final manuscript.
